# Supplementary material for: Robust all-optical single-shot readout of nitrogen-vacancy centers in diamond
Source: Nat Commun. 2021 Jan 22;12:532. doi: 10.1038/s41467-020-20755-3 (PMC7822820; doi:10.1038/s41467-020-20755-3)
Supplement: Supplementary file 1 — Supplementary Information [file 41467_2020_20755_MOESM1_ESM.pdf]

Supplementary Information for
“Robust All-Optical Single-Shot Readout of Nitrogen-Vacancy Centers in Diamond”

S.1 Hyperfine Structure and Magnetic Field Evaluation

Standard pulsed Optically Detected Magnetic Resonance (ODMR) techniques were employed for the evaluation of bias magnetic field strength and amplitude. By reducing the MW power used to excite the NV^- spin with a π-pulse, it is possible to selectively address the hyperfine energy levels resulting from the interaction between the NV^- electron spin and the ^{14}N nuclear spin $I = 1$ that forms the color center itself. The overall ground state Hamiltonian $\hat{\mathcal{H}}_g$ is:

$$\hat{\mathcal{H}}_g = \hbar \left(D_g \hat{S}_z^2 + \gamma_e \hat{S} \cdot \mathbf{B} + \hat{S}\hat{A}\hat{I} + Q_g \hat{I}_z^2 + \gamma_n \hat{I} \cdot \mathbf{B} \right).$$

The first term in the above Hamiltonian is dominant and can represent the system energy in the absence of external fields, i.e., is the zero-field term, where $\hbar$ is the reduced Planck constant. $\hat{S}$ is the electronic spin operator, and $D_g \simeq 2.87\,\mathrm{GHz}$ the axial zero-field parameter. In the following terms, $\mathbf{B}$ is the external bias magnetic field, $\gamma_e \simeq 2\pi \times 28\,\mathrm{MHz/mT}$ and $\gamma_n \simeq 2\pi \times -3.08\,\mathrm{kHz/mT}$ the electronic and nuclear gyromagnetic ratio, respectively, $Q = -4.945\,\mathrm{MHz}$ the nuclear quadrupole interaction, and $\hat{A}$ the hyperfine spin tensor with axial $A_\parallel = -2.16\,\mathrm{MHz}$ and orthogonal $A_\perp = -2.62\,\mathrm{MHz}$ components.

By solving the eigenvalue equation and considering the hyperfine transition energies as solution of the system, it is possible to determine the magnetic field amplitude B and orientation θ with respect to the NV quantization axis, the only unknown parameters. In this way, we measured $B = (0.7 \pm 0.1)\,\mathrm{mT}$ and $\theta = (39 \pm 7)^\circ$ for the deep NV center used in the main text. We want to stress that this was a non-optimized situation due to technical limitations and a first indication that the field alignment requirements are not strict. For the shallow NV center also studied in the main text, pulsed ODMR measurements were not able to resolve a more complex hyperfine structure, which highlight interactions with multiple nuclear spins related to other ^{13}C or N impurities. Nevertheless, since both the rough magnet position and the $|0\rangle \rightarrow |\pm 1\rangle$ transitions frequency range correspond to the case related to the deep NV, we can assume $B \leq 1\,\mathrm{mT}$, too.

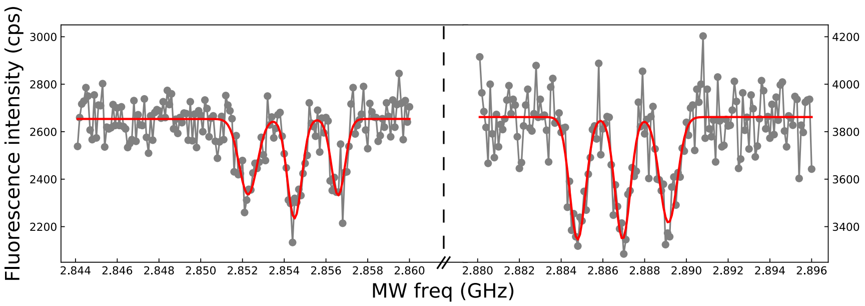

Figure S.1: Hyperfine ODMR transitions measured at room temperature. Grey scatter and red lines are experimental data and Lorentzian fit, respectively.

S.2 Simulation of the Excited State Structure and Optical Transitions

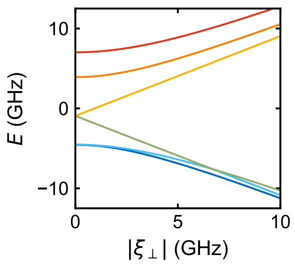

Figure S.2: Excited state structure of an NV^- center at low temperature depending on the non-axial strain. The magnetic flux density is 20 MHz and 45° misaligned. Coloring is for the readers convenience.

We simulated the NV^- excited state structure and the related optical transitions between ground and excited state according to Doherty *et al.*[1].

We solve the Hamiltonian as follows for 14 energy levels:

$$\mathcal{H} = V_{\text{opt}} + V_{\text{ss}} + V_{\text{so}} + \hat{O}_{E,x}(0,\xi_\perp) + \hat{S}_z + \hat{S}_x + \hat{S}_y$$

Here, V_{opt} is a diagonal matrix with the rough energy differences without considering structure within the ground and excited state. V_{ss} and V_{so} are the spin-spin and spin-orbit interaction potential according to Tab. 3 and Tab. 2 in Doherty *et al.*[1]. The values for the entries are taken from Tab. 4 in Doherty *et al.*[1]. $\hat{O}_{E,x}$ is an orbital operator according to Tab. A.4 in Doherty *et al.*[1], where we set the two different entries $O_{a,x} \rightarrow \frac{1}{\sqrt{2}} \langle a_1||V_E||e\rangle$ to 0 and $O_{b,x} \rightarrow \frac{1}{\sqrt{2}} \langle e||V_E||e\rangle$ to the non-axial strain $\xi_\perp$. $\hat{S}_{x,y,z}$ are the components of the total spin operator according to Tab. A.5 in Doherty *et al.*[1]. We set the entries S_i to the component of the magnetic flux density $\mathbf{B}$ in the "respective" direction. B_z is the component parallel to the NV center axis and determined as half the splitting from an ODMR spectrum.

The transition matrix element is a measure for the transition strength between the inital state $\mathbf{i}$ and the final state $\mathbf{f}$.

$$\mathcal{M}_{fi} = \left| \left\langle \mathbf{f} \middle\| \hat{O}_{E,x}(1,0) + \hat{O}_{E,y}(1,0) \middle\| \mathbf{i} \right\rangle \right|^2$$

Similarly to $\hat{O}_{E,x}$, $\hat{O}_{E,y}$ is an orbital operator according to Tab. A.4 in Doherty *et al.*[1], where we set the two entries $O_{a,y} \rightarrow \frac{1}{\sqrt{2}} \langle a_1||V_E||e\rangle$ to 1 and $O_{a,y} \rightarrow \frac{1}{\sqrt{2}} \langle e||V_E||e\rangle$ to 0.

For creating the simulated PLE spectra presented in Fig. 1d and 4a of the main text, we assumed Lorentzian broadening, so that the expected fluorescence is

$$f(E) = \sum_l \frac{A}{\sqrt{\pi\gamma}} \cdot \frac{\gamma^2}{\gamma^2 + (E - E_l)^2}$$

We set the amplitude A to $\mathcal{M}_{fi}$ and the FWHM γ to $\mathcal{M}_{fi}/10$. E_i is the energy of the different transitions, i.e. $(E_f - E_i)$.

In literature, the lower-energy spin $|0\rangle$ transition (E_y branch) is reported to have more spin mixing with the $|\pm1\rangle$ states and is accordingly less cycling.[2,3] This statement is typically made with the exception of low strain, which is the case of the used deep NV center, where we measured the lower-

energy transition to be more stable. This is in agreement with the simulation, where this transition is well separated in energy as well as the related excited state level (Fig. S.2 green line).

S.3 Saturation Curves

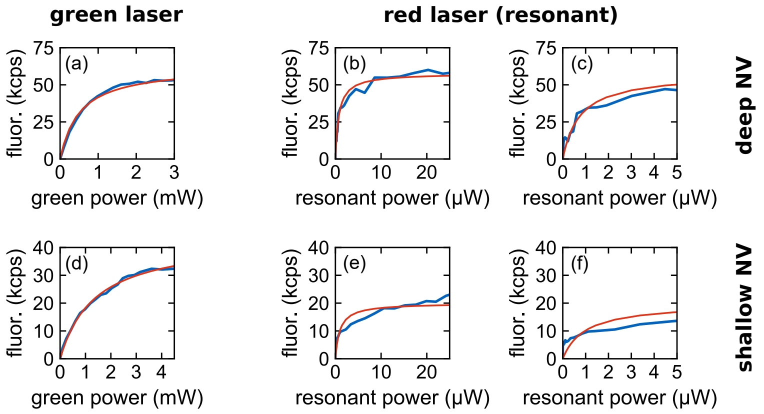

Figure S.3: Saturation curves for both NV centers presented in the main text. Both for illuminating them with a green 517 nm laser (a,d) and a narrow-band laser tuned to an NV^- spin $|0\rangle$ transition (b,e). Panels c and f are zoom-ins to the low-power regime of panels b and e, respectively. The red lines are fits to the data.

Table S.1: Overview of the saturation powers and the related saturation fluorescence for the data presented in Fig. S.3.

	$f_{\mathrm{sat,g}}$ (kcps)	$I_{\mathrm{sat,g}}$ (mW)	$f_{\mathrm{sat,r}}$ (kcps)	$I_{\mathrm{sat,r}}$ (μW)
deep NV	63 ± 6	0.51 ± 0.04	58 ± 10	0.74 ± 0.09
shallow NV	44 ± 3	1.38 ± 0.07	20 ± 333	1 ± 12

We measured the saturation behavior for both the deep NV center and the shallow NV center presented in the main text. For both NVs, we determined the saturation under illumination with a green 517 nm laser with 20 % duty cycle, where Fig. S.3a,d displays the duty-cycle corrected data. To measure the saturation behavior for the narrow-band red 637 nm laser tuned into resonance with a spin $|0\rangle$ transition (Fig. S.3b,c,e,f), we first initialized the NV centers charge and spin state with the green laser (without postselection). The actual illumination with the red laser is 100 ns short to acquire data without strong depolarization effects.

To determine the saturation power and fluorescence, we fitted the data with $f = A\frac{I \cdot I_{\mathrm{sat}}}{I + I_{\mathrm{sat}}}$.[4,5] $f_{\mathrm{sat}} = A \cdot I_{\mathrm{sat}}$.

For the deep NV center, the saturation count rate under resonant illumination is similar to the saturation value under green illumination. However, for the shallow implanted NV center, it differs a lot, and we cannot observe a clear saturation behavior when illuminating resonantly. We attribute this to spectral diffusion of the shallow implanted NV^- center's optical transition; increasing the laser power leads to power-broadening and in turn the laser line "hits" the optical transition more often. Note that in case of charge instability, increasing the resonant power is expected to shift the charge state balance more and more towards the dark NV^0 charge state because 637 nm illumination can

cause ionization to NV^0 but cannot cause recombination from NV^0. Hence, we exclude this effect as a source for the non-saturation behavior; in particular it is even a slight indication that the charge state seems to be reasonably stable in our case.

S.4 Speed-Up Factor

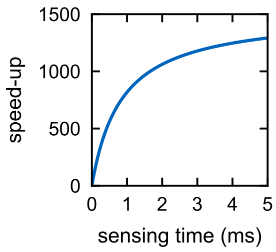

Figure S.4: Estimated speed-up when using the readout protocol presented in this manuscript in comparison with conventional fluorescence-based readout.

To determine the speed-up in comparison to the conventional off-resonant readout with a green laser, we estimated the time necessary to get an (average) SNR larger than one. For the single-shot protocol it is always only one repetition with length of 850 µs plus the time for the actual sensing sequence (e.g. a XY8 sequence). For the conventional readout, the single-shot SNR was calculated as described in Section S.11. The average SNR is $\sqrt{N} \cdot \mathrm{SNR}$, where N is the number of repetitions. Per repetition, we estimated 1.5 µs plus the actual sensing sequence.

Fig. S.4 shows the speed-up as function of the sensing sequence length. We assumed that the conventional readout can take advantage of the full saturation countrate of 50 kcps with a fluorescence contrast of 30 % for 250 ns between the different spin states. Note that the speed-up factor is already 2 for a zero-length sensing sequence.

As the presented protocol relies on low temperature, we also compare it with the resonant excitation readout method presented by Robledo *et al.*[8]. We measured histograms for both their method and our method in a higher-strain environment (~5.7 GHz) because strain has changed over time. To have the highest comparability possible experimental parameters were as similar as possible by three measures taken. 1) Both measurements were taken within an hour. 2) The resonant excitation readout was performed by just slightly modifying the pulse protocol used to measure our protocol: The ionization pulse was removed, and during the final (charge-state) readout the cw MW excitation was omitted to have a spin-dependent signal as used by Robledo *et al.*[8]. 3) All laser powers were kept to the same value, except for the final resonant excitation readout pulse, which was performed for different powers between 3 nW and 144 nW.

Our protocol resulted in an end-to-end spin readout fidelity of 83.8 % (SNR 1.38) for 1 ms readout duration and 79.4 % (SNR 1.04) for 200 µs readout duration. However, in our setup with a deep NV below a planer surface, the resonant excitation method performed much below an SNR of 1: the fidelity was only 52.8 % (SNR 0.22), best for 100 µs readout at 92 nW. Fig. S.5 presents the related count statistics. In turn, to get an average SNR of 1, 20 repetitions are necessary based on the single-shot SNR stated above.

Considering the same overhead of around 730 µs per repetition (mainly due to 500 µs for postselection)

for both protocols, our low-temperature spin-to-charge conversion promises to be faster by a factor of ∼20 independent of readout duration compared to the current state-of-the-art readout in low-temperature environments – provided low countrate, e.g. because of poor collection efficiency due to not using photonic structures.

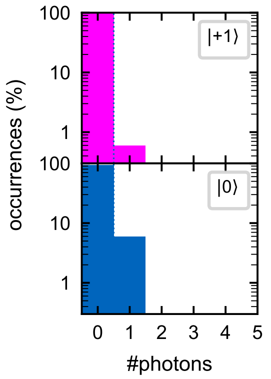

Figure S.5: Count statistics for the resonant excitation readout method according to Robledo *et al.*[8]. The count statistics was taken during 100 µs readout duration with the setup and sample geometry used throughout the rest of this publication; this includes in particular the absence of photonic structures.

S.5 Spin Initialization

A major limitation concerning both quantum sensing and quantum computing with NV centers is the NV^- spin state initialization. In this section, we present the spin dynamics under resonant optical pumping on an optical transition. First, we exploit the depletion of the related spin state for improving the overall spin initialization. Second, based on these time average measurements, we modeled the spin dynamics with a rate equation that allows to extract the population of each spin state.

S.5.1 Related Measurements

Commonly in NV center research, the NV center's charge and spin state is initialized into NV^- with spin $|0\rangle$ by illuminating it off-resonantly with a green laser (typically 532 nm). According to Doherty *et al.*[6], "the degree of ground state optical spin- polarisation [into spin $|0\rangle$] is not consistently reported in the literature, with many different values ranging from 42%–96% reported". Hopper *et al.*[7] report a value of around 80 %.

To determine the spin state distribution, we illuminated the NV center with the 637 nm laser at 56 nW tuned into resonance with an optical transition with $|0\rangle$ character. Doing this directly after initialization, the fluorescence intensity correlates with the spin $|0\rangle$ population. To get the actual $|0\rangle$ fraction, the same measurement is necessary for the spin $|\pm1\rangle$ populations. To access them, we swap the $|0\rangle$ population either with the $|+1\rangle$ or $|-1\rangle$ population by means of a MW π-pulse. As imperfect π-pulses can cause additional sources of errors, we also measure the fluoresce after two π-pulses on the MW $|+1\rangle$ transition. Fig. S.6a shows these four measurements for the deep NV center presented in the main text. The red lines are fits to the curve to determine the actual values, as discussed below. Except for the π-π-pulse curve and the fits, these data are presented in Fig. 2a in the main text, too.

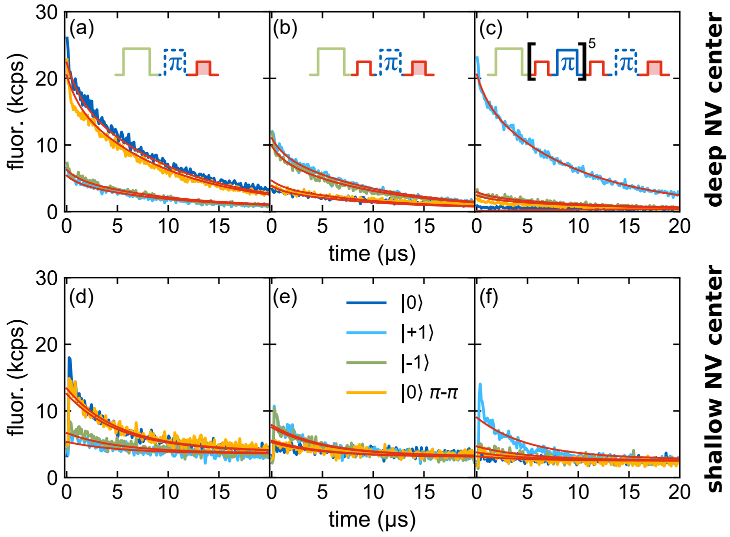

Figure S.6: Fluorescence during optical pumping on a spin $|0\rangle$ transition of the deep NV center (a-c) and the shallow NV center (d-f) used in the main text. Each panel shows four curves; either directly after optical initialization (dark blue), after a MW π-pulse on the $|+1\rangle$ and $|-1\rangle$ transition (light blue and green) as well as after two π-pulses on the $|+1\rangle$ transition (orange). In panels a and d, the spin was initialized with a green laser pulse; in b and e, with a green laser pulse followed by a resonant pulse; and in c and f by the full spin initialization protocol. See the insets for the measurement pulse sequences. All traces in each row (a-c; d-f) were measured interleaved. For better distinguishability, the measurement data are averaged over ten bins, with each bin of 10 ns being the average of 500 000 (200 000) experimental repetitions for the deep (shallow) NV center. The red lines are fits to the non-averaged data, see Section S.5.2.

Fig. S.6b displays the spin distribution after 20 µs illumination with the resonant laser; that is the spin distribution after the first measurement without any MW excitation. Similarly to Fig. S.6a, the different spin populations are measured by swapping the population to spin zero by MW π-pulses. In accordance with Robledo *et al.*[8], the first pulse has mainly depleted the spin $|0\rangle$ transition and in turn spin $|\pm1\rangle$ is much higher populated.

To exploit this for higher selectivity in spin initialization, after the first resonant pulse we swap the slightly lower-occupied $|-1\rangle$ population back to $|0\rangle$ by a MW π-pulse. Repeating this several times, more and more population accumulates in the other spin one state $|+1\rangle$, as can be seen in Fig. S.6c.

Fig. S.6d-f present the same measurements for the shallow NV center used in the main text. The pronounced difference is the curves not decaying to zero but to a finite value. This stems from simultaneously also exciting an optical $|\pm1\rangle$ transition, which is spectrally close to the used optical $|0\rangle$ transition (compare Fig. 4a in the main text). This has implications on the used sequence for spin polarization: As longer illumination will mix the spin populations more and more, we shortened each optical pumping pulse to 10 µs. The resonant laser was operated at 170 nW, which is the power, where just a slight decrease in fluorescence happens during resonant illumination together with cw MW spin mixing, as used for the charge-state readout.

S.5.2 Rate Equation Model

To determine the spin-state distribution and in turn the spin-initialization fidelity, we modeled the NV center dynamics as rate equations with five states. The states are 1) NV^- $|0\rangle$, 2) NV^- $|+1\rangle$, 3) NV^- $|-1\rangle$, 4) NV^- singlet and 5) NV^0.

$$n = \begin{pmatrix} n_{|0\rangle} \\ n_{|+1\rangle} \\ n_{|-1\rangle} \\ n_{\mathrm{sing}} \\ n_{\mathrm{NV}^0} \end{pmatrix}$$

n_i is the time-average population of the NV center being in state i. At the beginning of each fit, the populations of the three different spin states add to one, while the probabilities to be in the singlet or in NV^0 are set to 0.

The dynamics while pumping on an optical transition with spin $|0\rangle$ character is modelled with the transfer matrix as follows:

$$T_{\mathrm{opt}} = \begin{pmatrix} & 0 & 0 & p_{\mathrm{ts}}/2 & 0 \\ 0 & & 0 & p_{\mathrm{ts}}/4 & 0 \\ 0 & 0 & & p_{\mathrm{ts}}/4 & 0 \\ p_{\mathrm{st},0} & p_{\mathrm{st},1} & p_{\mathrm{st},1} & & 0 \\ p_{\mathrm{ion}} & p_{\mathrm{ion}} & p_{\mathrm{ion}} & 0 & \end{pmatrix}$$

To maintain probabilities, the empty diagonal entries are 1 minus the sum of the other elements in the respective column; e.g. the 5th diagonal element is 1. $p_{\mathrm{st},0}$ and $p_{\mathrm{st},1}$ are the probabilities of having an inter-system crossing to the singlet in one time step, starting from spin $|0\rangle$ or $|\pm1\rangle$, respectively. p_{ts} is the probability of an inter-system crossing back to the triplet, where we assumed that $\frac{1}{2}$ of events end up in spin $|0\rangle$ and the other half is equally distributed to both $|\pm1\rangle$ states. Finally, p_{ion} is the probability of ionizing the NV^- center to NV^0 within one measurement time bin. We do not include recombination from NV^0 to NV^- because of the 637 nm laser's energy per photon and its low intensity render this process very unlikely.

The excited state is excluded in the model, too, as it is implicitly described by the fluorescence parameters f_0 and f_1. These are used as link to fit the measured fluorescence decay curves, where the total fluorescence was modelled as follows for each time step:

$$f(t) = n_{|0\rangle}(t) \cdot f_0 + n_{|+1\rangle}(t) \cdot f_1 + n_{|-1\rangle}(t) \cdot f_1$$

The MW π-pulses are modeled as follows (here for a π-pulse on the $|+1\rangle$ transition):

$$T_{\mathrm{MW},+1} = \begin{pmatrix} E_{\mathrm{MW}} & 1-E_{\mathrm{MW}} & 0 & 0 & 0 \\ 1-E_{\mathrm{MW}} & E_{\mathrm{MW}} & 0 & 0 & 0 \\ 0 & 0 & 1 & 0 & 0 \\ 0 & 0 & 0 & 1 & 0 \\ 0 & 0 & 0 & 0 & 1 \end{pmatrix}$$

As the overall fluorescence lowers after each optical pumping step (compare Fig. S.6a/d → b/e → c/f), we also include a "fluorLoss" parameter, which accounts for the reduced overall fluorescence in Fig. S.6b,e (fluorLoss1) as well as d,f (fluorLoss6). We attribute this loss of fluorescence mainly to ionization at the very first resonant illumination, when the spin $|0\rangle$ population is high. In turn, there

is a high population in the NV$^-$ excited state, which can get ionized comparably easy even by the low-power 637 nm laser.

Fig. S.7 shows all 12 measured fluorescence time traces under optical pumping. Tab. S.2 summarizes the values from fitting all 12 curves simultaneously with the rate equation model described above.

Table S.2: NV$^-$ spin state distribution according to fitting with the rate equation model described in Sec. S.5.2 (upper two sub-tables) and global fitting parameters for all panels in Fig. S.7 together (third sub-table). In the upper two sub-tables, a-f refer to the panels in Fig. S.6 and S.7. The empty cell denote a lifetime that is several orders of magnitude larger, in the range of hours. Lifetimes ($1/e$) are calculated as $T_i = -\text{binwidth}/\ln(1-p_i)$.

deep NV			
	a	b	c
$n_{\|0\rangle}$	70.4 ± 1.2	14.8 ± 0.6	0.0 ± 1.1
$n_{\|+1\rangle}$	13.4 ± 0.6	45.1 ± 0.4	87.9 ± 1.7
$n_{\|-1\rangle}$	16.3 ± 1.3	40.1 ± 0.7	12.1 ± 2.0
$\mathcal{F}_{\text{spin}}$	85.2 ± 0.6	72.6 ± 0.2	94.0 ± 0.9

shallow implanted NV			
	d	e	f
$n_{\|0\rangle}$	70.4 ± 1.8	18.9 ± 0.9	6.9 ± 1.4
$n_{\|+1\rangle}$	9.7 ± 1.2	42.1 ± 0.8	70.0 ± 1.9
$n_{\|-1\rangle}$	19.8 ± 2.2	38.9 ± 1.2	23.1 ± 2.3
$\mathcal{F}_{\text{spin}}$	85.2 ± 0.9	71.1 ± 0.4	85.0 ± 0.9

	deep NV	shallow NV
E_{MW} (%)	5.6 ± 0.1	5.1 ± 0.6
f_0 (kcps)	31.7 ± 0.6	17.5 ± 0.4
f_1 (kcps)	0.2 ± 0.3	3.6 ± 0.2
$T_{\text{st},0}$ (µs)	4.1 ± 0.3	7.0 ± 0.7
$T_{\text{st},1}$ (ms)	0.4 ± 0.3	1.0 ± 10.0^{-6}
T_{ts} (µs)	1.33 ± 0.09	11.3 ± 2.9
T_{ion} (ms)		0.2 ± 0.6
fluorLoss1 (%)	20.5 ± 0.3	15.1 ± 0.5
fluorLoss6 (%)	21.9 ± 0.3	30.0 ± 0.5
R^2	0.944	0.537

S.6 Charge Initialization

The NV charge state was initialized with a green 517 nm laser pulse of 2 µs length at 1.4 mW in case of the deep NV center. The initialization was optimized as follows: After an ionization pulse, different initialization powers and times were applied, followed each by a charge readout step. The initialization (without postselection) that maximized the average fluorescence during the readout step was used.

For the shallow NV center, we used the initialization sequence as follows: red 642 nm laser at

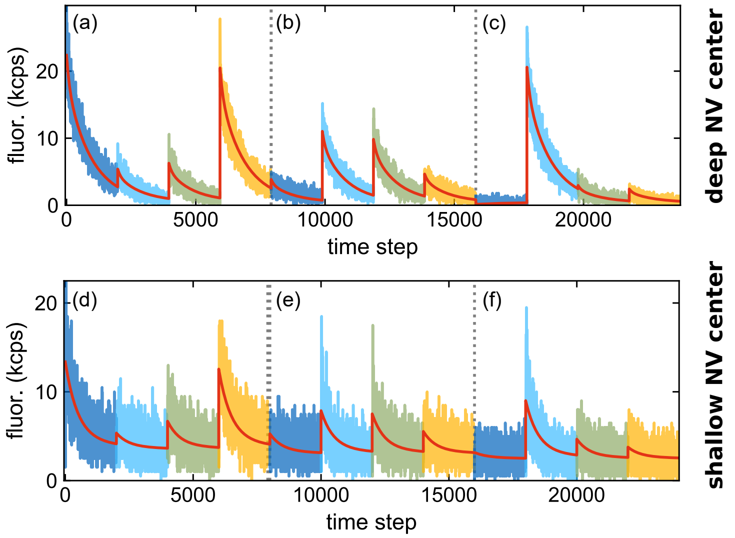

Figure S.7: Fit of the fluorescence decay curves under optical pumping. Same data than in Fig. S.6, including the same color code. Here each measurement bin is shown, as used for the fitting. Each row displays the trace as measured, i.e. the 12 different "sub-curves" were measured as a whole before the next repetition of all "sub-curves". To minimize effects related to the previous sequence, an additional green (2 µs) and resonant (20 µs) pulse was applied, before next the "sub-curve" starts with the respective initialization.

>17 mW for 1 µs → 500 ns break → green 517 nm laser at 3.6 mW for 3 µs → 200 ns break → green 517 nm laser at 3.6 mW for 200 ns. This performed better than a single green initialization pulse with respect to the average fluorescence related to a charge readout afterwards (see above for the optimization procedure). We attribute this to changing the local surrounding of the shallow implanted NV center. Such changes were demonstrated to shift the charge-state balance.[9]

To improve the charge initialization fidelity for the final measurements, we applied a charge readout step of 500 µs (1 ms) directly after the initialization sequence for the deep (shallow implanted) NV center and postselected on events with at least 6 (2) photons, which corresponds to an acceptance rate of about 37 % (22 %) of repetitions.

To quantify the charge initialization and readout, we measured the photon count statistics for a charge readout both directly after the initialization and after a strong ionization pulse in between. This ionization pulse was 5 µs of resonant laser together with the 642 nm laser, followed by 15 µs with additional cw MW to counteract depolarization. In this supplementary information, we present the data taken for long photon acquisition time as this promises the least error due to charge readout and in turn is the best estimate for the charge initialization to NV^-. The resulting NV^- and NV^0 count statistics is presented in Fig. S.8, which includes fits with Poisson and Gauß distributions. To distinguish between both charge states, we set a threshold that was determined by minimizing the sum of errors for both distributions, i.e. the percentage of events below (above) threshold for the NV^- (NV^0) distribution.

For the deep NV center, the NV^0 distribution for 5 ms readout duration can be fitted well ($R^2 = 0.999\,96$) with a Poison distribution centered around (0.4712 ± 0.0004) photons. The NV^- distribution can be fitted ($R^2 = 0.927$) with the sum of two Gaussians. We attribute the Gaussian nature to spectral diffusion of the deep NV center's optical transitions, so that the spectral overlap between the laser

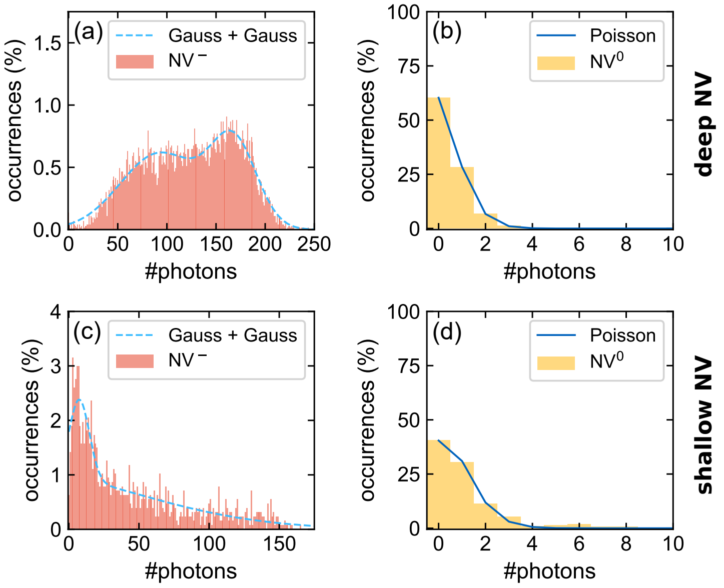

Figure S.8: Count statistics for the charge-state readout. For the deep NV center (a,b) the readout time was 5 ms and for the shallow implanted NV center (c,d) it was 10 ms. The left column displays the distribution after the charge-state initialization, while the right column has a strong ionization pulse included between initialization and readout.

mode and the NV transition varies from repetition to repetition. The NV^- distribution has 0.25 % of events below threshold (5 photons), which is considered as NV^0 fraction after the charge initialization.

For the shallow NV center, the distributions were evaluated at the maximum readout time of 10 ms. Here, the fraction of the NV^- distribution below threshold (4 photons) is 7.1 %. The NV^0 distribution can be fitted well with a Poissonian ($R^2 = 0.995$) around (0.766 ± 0.006) photons. The NV^- distribution has to be fitted again with the sum of two Gaussians ($R^2 = 0.87$). They are centered around (-46 ± 75) and (7.6 ± 0.6) photons with a standard deviation of (65 ± 19) and (5.0 ± 0.6) photons. The amplitudes are (1.1 ± 0.6) and (1.5 ± 0.1) %.

To determine the threshold for the spin-state distribution, we minimized the charge-state error as described above. The only difference is that we used the same readout time than for the spin-state distribution; compare Fig. 2d and 3b in the main text, which were both acquired with 1 ms of readout time.

S.7 Ionization with NIR

According to simple energy considerations, the second step for the NV^- ionization might be possible with infrared (IR). The ionization energy for NV^-, i.e. the energy difference between the NV^- ground state and the conduction band of diamond, was reported to be 2.60 eV[5]. By just subtracting 1.95 eV (637 nm; zero-phonon line of NV^-) from the ionization energy, even a wavelength as long as 1900 nm might serve as second photon for the ionization.

To ionize a deep NV center, we compared using a red 642 nm and an IR 980 nm laser, which both should serve as source for the 2nd photon. Both lasers are pigtailed laser diodes with single-mode fiber. Fig. S.9 presents measurements similar to Fig. 3a and 4c in the main text. We do see a reduction in

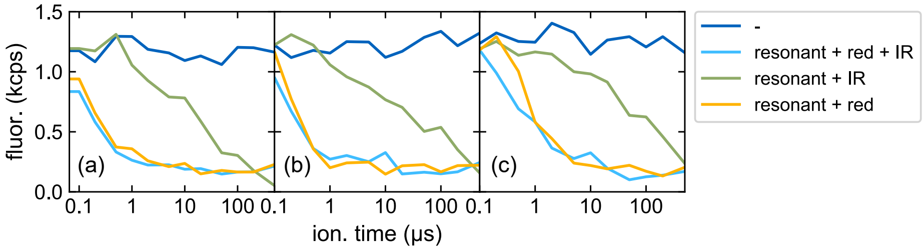

Figure S.9: Fluorescence after ionization with different lasers and powers. The NV center was either subjected to no ionization (dark blue), to a combined pulse of the resonant + red + IR laser (light blue), resonant + IR (green) and resonant + red (orange). The lasers were either (a) at their maximum powers (red at 17 mW and IR at 33 mW), (b) at 17 mW and (c) at 10 mW. All traces within each panel were taken interleaved.

fluorescence when illuminating the NV center simultaneously with the resonant laser (which provides the first photon to excite the NV^-) and the IR laser (providing the second photon to ionize the NV^-). However, compared to the red 642 nm laser, the ionization time needs to be more than one order of magnitude longer, indicating a much worse absorption cross section for 980 nm. We used the red 642 nm laser as source for the second photon for all other data presented in this manuscript.

S.8 Correcting the Readout Fidelity

The spin fidelity as measured in Fig. 3b and 4d of the main text is a measure of the end-to-end performance of the whole protocol. This includes the spin-dependent ionization and the readout step itself, but also non-perfect charge and spin initialization as well as errors of the MW. These additional error sources due to initialization and MW are independent of the readout that is chosen and depend on the overall technical implementation. To correct for these effects and to get the actual fidelity related to just our readout scheme, we model the whole protocol as a multi-step process, see Fig. S.10.

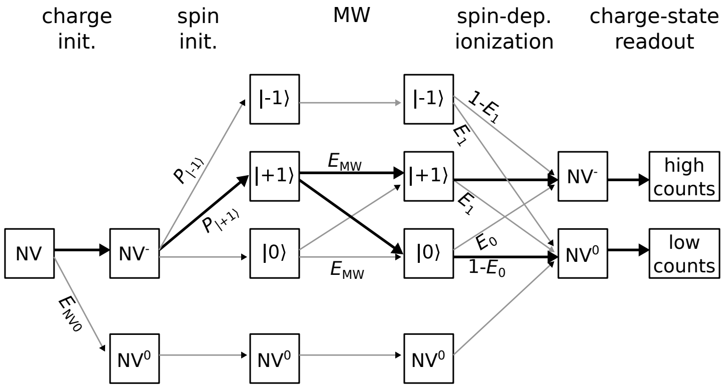

Figure S.10: Model of the whole protocol. Steps are charge and spin initialization, MW π pulse (or no MW), spin-dependent ionization and the final charge-state readout.

Here, all variables are probabilities, which means that at any branching, the different leaving paths add up to 1. E_{NV0} is the population in the neutral NV^0 charge state after charge initialization (including postselecting on the charge state). $P_{|-1\rangle}$ and $P_{|+1\rangle}$ are the probabilities of having spin $|-1\rangle$

and $|+1\rangle$, respectively. Accordingly, the probability of initializing into spin $|0\rangle$ is $(1 - P_{|-1\rangle} - P_{|+1\rangle})$. E_{MW} is the error of a MW π-pulse, meaning that with this probability, the π pulse does not flip the spin. E_0 is the error of not ionizing spin $|0\rangle$ despite having tuned the resonant laser to a $|0\rangle$ transition, and E_1 the error of ionizing spin $|\pm 1\rangle$. The bold black arrows indicate the paths for perfect experimental conditions. This includes the explicit spin initialization into $|+1\rangle$, which was used for the spin-dependent count statistics presented in Fig. 3b (4d) for the deep (shallow) NV center.

It is important to note, that the actual fidelity of our readout protocol just refers to the steps 'spin-dependent ionization' and 'charge-state readout', which are modeled together with the parameters E_0 and E_1. On the other side, the errors that are used to determine the as-measured end-to-end fidelity are $E_{0,\mathrm{meas}}$ and $E_{1,\mathrm{meas}}$. $E_{0,\mathrm{meas}}$ ($E_{1,\mathrm{meas}}$) is the sum of all paths that end up in high (low) counts, while intending to prepare the spin in $|0\rangle$ ($|+1\rangle$) by means of a MW π-pulse (no MW π-pulse) in step 3 after the spin initialization.

Summing up all paths, we get a linear equation system with two unknown variables E_0 and E_1.

$$\begin{aligned}
E_{0,\mathrm{meas}} = {} & (1 - E_{\mathrm{NV0}}) \cdot P_{|-1\rangle} \cdot (1 - E_1) \\
+ {} & (1 - E_{\mathrm{NV0}}) \cdot P_{|+1\rangle} \cdot E_{\mathrm{MW}} \cdot (1 - E_1) \\
+ {} & (1 - E_{\mathrm{NV0}}) \cdot P_{|+1\rangle} \cdot (1 - E_{\mathrm{MW}}) \cdot E_0 \\
+ {} & (1 - E_{\mathrm{NV0}}) \cdot (1 - P_{|-1\rangle} - P_{|+1\rangle}) \cdot (1 - E_{\mathrm{MW}}) \cdot (1 - E_1) \\
+ {} & (1 - E_{\mathrm{NV0}}) \cdot (1 - P_{|-1\rangle} - P_{|+1\rangle}) \cdot E_{\mathrm{MW}} \cdot E_0
\end{aligned}$$

$$\begin{aligned}
E_{1,\mathrm{meas}} = {} & (1 - E_{\mathrm{NV0}}) \cdot P_{|-1\rangle} \cdot E_1 \\
+ {} & (1 - E_{\mathrm{NV0}}) \cdot P_{|+1\rangle} \cdot E_1 \\
+ {} & (1 - E_{\mathrm{NV0}}) \cdot (1 - P_{|-1\rangle} - P_{|+1\rangle}) \cdot (1 - E_0) \\
+ {} & E_{\mathrm{NV0}}
\end{aligned}$$

The variables and solutions for the deep and shallow NV center are summarized in Tab. S.3.

Table S.3: Overview of the measured end-to-end spin fidelity $\mathcal{F}_{\mathrm{meas}}$ as well as the probabilities/errors that were estimated for the initialization with the explicit spin initialization and for the MW. These values were taken into account when solving the linear equation system with solutions E_0 and E_1. The charge initialization was assumed to be perfect. Photons were collected for 1 ms (10 ms) for the deep (shallow) NV center.

	deep NV	shallow NV
$E_{0,\mathrm{meas}}$ (%)	17.6 ± 0.7	44.3 ± 1.3
$E_{1,\mathrm{meas}}$ (%)	5.4 ± 0.7	21.4 ± 1.3
$\mathcal{F}_{\mathrm{meas}}$ (%)	88.5 ± 0.5	67.1 ± 0.9
$P_{\vert-1\rangle}$ (%)	12.1 ± 2.0	23.1 ± 2.3
$P_{\vert+1\rangle}$ (%)	87.9 ± 1.7	70.0 ± 1.9
E_{MW} (%)	5.6 ± 0.1	5.1 ± 0.6
E_0 (%)	1.8 ± 2.2	25.3 ± 3.1
E_1 (%)	5.4 ± 2.5	17.4 ± 2.5
$\mathcal{F}$(%)	96.4 ± 2.2	78.6 ± 2.5
single-shot SNR	3.5 ± 1.2	0.99 ± 0.13

S.9 Photon Collection Statistics for Short Readout

Table S.4: Readout metrics for different readout times. End-to-end charge and spin fidelities of the deep NV center presented in the main text, as well as fidelities corrected by spin-initialization and MW imperfections. The data for 1 ms refer to the data presented in the main text. Note that the charge count statistics are not taken into account for correcting the spin fidelity—it is presented here to have a more clear estimate on the influence of the charge readout fidelity.

	10 ms	1 ms	100 µs	50 µs
E_{NV0} w/o post-sel. (%)	46.1 ± 0.7	49.0 ± 0.7	60.0 ± 0.7	70.9 ± 0.7
E_{NV0} (%)	0.3 ± 0.7	0.4 ± 0.7	14.9 ± 0.7	35.0 ± 0.7
threshold (photons)	6	3	1	1
$\mathcal{F}_{\mathrm{charge,meas}}$ (%)	98.2 ± 0.5	98.1 ± 0.5	85.3 ± 0.5	76.8 ± 0.5
$E_{0,\mathrm{meas}}$ (%)	17.7 ± 0.7	17.6 ± 0.7	16.2 ± 0.7	12.2 ± 0.7
$E_{1,\mathrm{meas}}$ (%)	5.2 ± 0.7	5.4 ± 0.7	19.2 ± 0.7	37.3 ± 0.7
$\mathcal{F}_{\mathrm{meas}}$ (%)	88.5 ± 0.5	88.5 ± 0.5	82.3 ± 0.5	75.2 ± 0.5
E_0 (%)	1.9 ± 2.2	1.8 ± 2.2	2.9 ± 1.9	1.8 ± 1.6
E_1 (%)	5.2 ± 2.5	5.4 ± 2.5	19.2 ± 2.2	37.7 ± 1.7
$\mathcal{F}$(%)	96.5 ± 0.2	96.4 ± 2.2	88.9 ± 1.8	80.4 ± 1.5
single-shot SNR	3.6 ± 1.2	3.5 ± 1.2	1.8 ± 0.2	1.21 ± 0.10

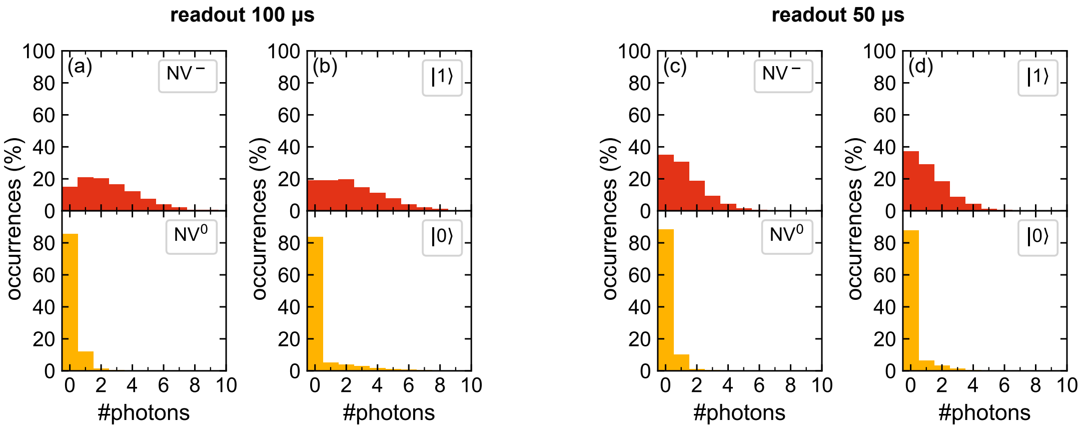

Figure S.11: Count statistics for 100 µs and 50 µs readout duration with the deep NV center presented in the main text. Parts a,c (b,d) were measured simultaneously with the data presented in Fig. 2d (3b) in the main text.

The charge and spin count statistics (histograms) of the readout presented in Fig. 2d and 3b were measured with an actual readout duration of 10 ms. However, the software measured count statistics not only for the whole 10 ms, but also after the first 0.05, 0.1, 0.2, 0.5, 1, 2 and 5 ms. (In the main text, they were evaluated for 1 ms.) In the first place, this allows to compare different readout times for otherwise exact same measurement parameters. In particular, it indicates that for the already quite low saturation count rate of 50 kcps for this NV center in this setup we can speed up the measurement a lot by reducing the readout time to 100 µs, while still maintaining an end-to-end fidelity of $> 79\,\%$, which corresponds to a single-shot SNR $> 1^7$. Tab. S.4 summarizes the end-to-end fidelities for readout durations spanning two orders of magnitude and Fig. S.11 presents some related count statistics.

In the second place, this simultaneous acquisition of count statistics for different readout times allows to mimic the situation for poor collection optics: Evaluating just the first 100 µs of readout still gives a good directly measured end-to-end fidelity of 82.3 %. Together with the fluorescence being stable over the whole 10 ms of laser illumination, it resembles the situation of $1/100^{\text{th}}$ the detected fluorescence count rate and taking the count statistics for the whole 10 ms. With the NV center used for taking these data having a saturation count rate of 50 kcps, a single-shot readout would be possible even for a saturation count rate as low as 500 clicks per second.

S.10 Spectral Diffusion

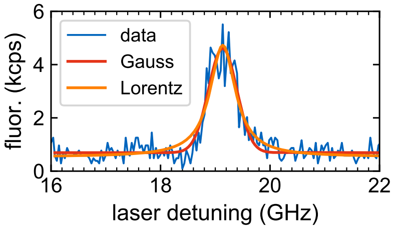

Figure S.12: Fitting the broadened optical transition. Section of the PLE spectrum presented in Fig. 4a in the main text. The used optical transition was fitted both with a Gaussian and Lorentzian curve in this section.

To determine to which extent the PLE linewidth of the shallow implanted NV used in the main text is limited by spectral diffusion, we fitted the used $|0\rangle$ transition both with a Gaussian and a Lorentzian curve (Fig. S.12). While the Lorentzian fit has an R^2 of 0.69, the Gaussian fit performs better with an R^2 of 0.87. This indicates that inhomogeneous broadening due to spectral diffusion is the main reason for broadening, with a Gaussian FWHM of (0.43 ± 0.02) GHz. Note that the initialization procedure for this measurement was different with green illumination for 500 ns at 1.2 mW. In contrast, the initialization in the other measurements with the shallow NV center include a longer (3 µs) and stronger (3.6 mW) green pulse and in addition a >17 mW red pulse for 1 µs. Thus, the inhomogeneously broadened linewidth in the relevant measurements is expected to be even broader.

S.11 SNR and Fidelity Calculation

The numbers for the single-shot SNR and the fidelity are calculated according to Hopper *et al.*[7]. For the common off-resonant readout with a green laser, the single-shot SNR is estimated as

$$\mathrm{SNR} = \frac{\#ph - 0.7 \cdot \#ph}{\sqrt{\#ph + 0.7 \cdot \#ph}}$$

where $\#ph = f_{\text{sat}} \cdot 250\,\text{ns}$ is the photon number per readout repetition and f_{sat} is the saturation count rate when illuminating the NV center with a green laser. We assume the contrast between spin states to be 0.3.

For our protocol, we use thresholding to separate between the charge states and in turn the spin

states. The single-shot SNR was estimated as

$$\mathrm{SNR} = \frac{1 - E_1 - E_0}{\sqrt{(1 - E_1) \cdot E_1 + (1 - E_0) \cdot E_0}}.$$

References

1. Doherty, M. W., Manson, N. B., Delaney, P. & Hollenberg, L. C. L. The negatively charged nitrogen-vacancy centre in diamond: the electronic solution. *New Journal of Physics* **13,** 025019 (2011).

2. Tamarat, P., Manson, N. B., Harrison, J. P., McMurtrie, R. L., Nizovtsev, A., Santori, C., Beausoleil, R. G., Neumann, P., Gaebel, T., Jelezko, F., Hemmer, P. & Wrachtrup, J. Spin-flip and spin-conserving optical transitions of the nitrogen-vacancy centre in diamond. *New Journal of Physics* **10,** 045004 (2008).

3. Batalov, A., Jacques, V., Kaiser, F., Siyushev, P., Neumann, P., Rogers, L. J., McMurtrie, R. L., Manson, N. B., Jelezko, F. & Wrachtrup, J. Low Temperature Studies of the Excited-State Structure of Negatively Charged Nitrogen-Vacancy Color Centers in Diamond. *Physical Review Letters* **102,** 195506 (2009).

4. Visscher, K., Brakenhoff, G. J. & Visser, T. D. Fluorescence saturation in confocal microscopy. *Journal of Microscopy* **175,** 162–165 (1994).

5. Aslam, N., Waldherr, G., Neumann, P., Jelezko, F. & Wrachtrup, J. Photo-induced ionization dynamics of the nitrogen vacancy defect in diamond investigated by single-shot charge state detection. *New Journal of Physics* **15,** 013064 (2013).

6. Doherty, M. W., Manson, N. B., Delaney, P., Jelezko, F., Wrachtrup, J. & Hollenberg, L. C. The nitrogen-vacancy colour centre in diamond. *Physics Reports* **528,** 1–45 (2013).

7. Hopper, D., Shulevitz, H. & Bassett, L. Spin Readout Techniques of the Nitrogen-Vacancy Center in Diamond. *Micromachines* **9,** 437 (2018).

8. Robledo, L., Childress, L., Bernien, H., Hensen, B., Alkemade, P. F. A. & Hanson, R. High-fidelity projective read-out of a solid-state spin quantum register. *Nature* **477,** 574–578 (2011).

9. Dhomkar, S., Jayakumar, H., Zangara, P. R. & Meriles, C. A. Charge Dynamics in near-Surface, Variable-Density Ensembles of Nitrogen-Vacancy Centers in Diamond. *Nano Letters* **18,** 4046–4052 (2018).
